# Weak immunogenicity of SARS-CoV-2 vaccine in patients with hematologic malignancies

**DOI:** 10.1038/s41408-021-00534-z

**Published:** 2021-08-10

**Authors:** Florent Malard, Béatrice Gaugler, Joel Gozlan, Lucie Bouquet, Djeneba Fofana, Lama Siblany, Deborah Eshagh, Olivier Adotevi, Caroline Laheurte, Laure Ricard, Rémy Dulery, Nicolas Stocker, Zoe van de Wyngaert, Alexis Genthon, Anne Banet, Mara Memoli, Souhila Ikhlef, Simona Sestilli, Anne Vekhof, Eolia Brissot, Zora Marjanovic, Yannick Chantran, Nancy Cuervo, Eric Ballot, Laurence Morand-Joubert, Mohamad Mohty

**Affiliations:** 1 APHP, Hôpital Saint Antoine, Service d’Hématologie Clinique et de Thérapie cellulaire, Paris, France; 2 Sorbonne Université, INSERM UMR938, Centre de Recherche Saint-Antoine (CRSA), F-75012 Paris, France; 3 APHP, Hôpital Saint Antoine, Department of Virology, Paris, France; 4 Department of Medical Oncology, University Hospital of Besançon, INSERM, EFS BFC, UMR1098 RIGHT, Université de Bourgogne Franche-Comté, Besançon, France; 5 Sorbonne Université Institut Pierre Louis d’Epidémiologie et de Santé Publique, INSERM UMR S1136, Paris, France; 6 APHP, Hôpital Saint Antoine, Service d’Immunologie, Paris, France

**Keywords:** Translational research, Haematological cancer, Adaptive immunity

## Abstract

This study evaluated the safety and immunogenicity of BNT162b2 vaccine in patients with hematological malignancies. Antibodies blocking spike binding to immobilized ACE-2 (NAb) correlated with anti-Spike (S) IgG d42 titers (Spearman r = 0.865, *p* < 0.0001), and an anti-S IgG d42 level ≥3100 UA/mL was predictive of NAb ≥ 30%, the positivity cutoff for NAb (*p* < 0.0001). Only 47% of the patients achieved an anti-S IgG d42 level ≥3100 UA/mL after the two BNT162b2 inocula, compared to 87% of healthy controls. In multivariable analysis, male patients, use of B-cell targeting treatment within the last 12 months prior to vaccination, and CD19^+^ B-cell level <120/uL, were associated with a significantly decreased probability of achieving a protective anti-S IgG level after the second BNT162b2 inoculum. Finally, using the IFN-γ ELISPOT assay, we found a significant increase in T-cell response against the S protein, with 53% of patients having an anti-S IgG-positive ELISPOT after the second BNT162b2 inoculum. There was a correlation between the anti-S ELISPOT response and IgG d42 level (Spearman r = 0.3026, *p* = 0.012). These findings suggest that vaccination with two BNT162b2 inocula translates into a significant increase in humoral and cellular response in patients with hematological malignancies, but only around half of the patients can likely achieve effective immune protection against COVID-19.

## Introduction

The dismal prognosis after infection by the severe acute respiratory syndrome coronavirus-2 (SARS-CoV-2), called Coronavirus Disease 2019 (COVID-19) in patients with hematologic malignancies is now well established. A meta-analysis, including 3377 adult patients with these diseases, reported a 34% risk of death in those suffering from COVID-19 [1]. Furthermore, B-cell depleting immunotherapy has been associated with prolonged in-hospital stay and higher mortality after COVID19 [2]. Therefore, patients with hematologic malignancies have been prioritized for primary prevention of COVID-19 with vaccination.

Based on randomized phase III clinical trials, several COVID-19 vaccines became available in late 2020, early 2021, with the BNT162b2 (Pfizer/BioNTech), ChAdOx1 nCoV-19 (Oxford/AstraZeneca), and mRNA-1273 (Moderna) vaccines being available in Europe and in the United States of America [3–5]. Nevertheless, people with hematologic malignancies were not included in those vaccine clinical trials. Hematologic malignancies are often associated with immunosuppression, and the treatment used can further worsen immune defect, raising the question of vaccine immunogenicity in those patients. In particular, anti-CD20 monoclonal antibodies used in lymphoid malignancies and other B-cell targeting treatment such as drugs used in multiple myeloma, including proteasome inhibitors, immunomodulatory drugs, anti-CD38 monoclonal antibodies, or steroids, may impair SARS-CoV-2 neutralizing antibody production after vaccination. Similarly, hematopoietic cell transplantation (HCT) is associated with a profound immune defect of both B and T cells [6], which raises the question of vaccine immunogenicity in those patients. In fact, it was recently reported in patients with chronic inflammatory disease that glucocorticoids and B-cell depleting agents substantially impair immunogenicity of mRNA vaccines to COVID19 [7].

In patients with hematologic malignancies, preliminary studies reported a low seroconversion rate after the first BNT162b2 inoculum in patients, ranging from 18 to 25% [8, 9]. These findings compare poorly with the results of a phase 1 mRNA vaccine immunogenicity trial that reported robust antibody responses after two injections of 30 μg of BNT162b2 in essentially 100% of healthy volunteers [10]. Therefore, it appears indispensable to evaluate the global vaccine immunogenicity in patients with hematologic malignancies, to determine the factors associated with the immune response and to decipher the mechanism underlying the lack of response in some patients, in order to adapt the vaccination strategy. Other key questions are whether, in patients with hematologic malignancies, antibodies from vaccine responders are able to functionally neutralize SARS-CoV-2 and if the T-cell response against SARS-CoV-2 epitopes is maintained.

With this background, we evaluated the safety and immunogenicity of BNT162b2 vaccine in the first 239 patients with hematologic malignancies vaccinated in our department.

## Patients and methods

### Patients

From 18 January 2021, patients with a history of allogeneic (allo) HCT and/or an active hematologic malignancy became eligible for COVID-19 vaccination in France. They were initially scheduled to receive two intramuscular injections of 30 μg of BNT162b2, 3 weeks apart. Based on the French health authority guidelines, the two injections were finally administered 4 weeks apart in most patients. Retrospective chart review was performed according to institutional guidelines. Written informed consent was obtained in accordance with the principles of the declaration of Helsinki.

### Safety assessment

Side effects after the first and second BNT162b2 injections were reviewed from patients’ charts and graded according to the Common Terminology Criteria for Adverse Events (CTCAE v5.0). Patients with symptoms suggesting a possible COVID-19 infection underwent a nasopharyngeal swab testing for SARS-CoV-2.

### Serological assessment

As part of routine monitoring practice, two serological assays were realized, one before the first and second vaccinations, on day 28, and another one 14 days after the second vaccination (day 42). Immunogenicity was assessed by automated chemiluminescence assay (CMIA), using both the SARS-CoV-2 reagents (Abbott, Rungis, France) for qualitative detection of anti-N IgG (aimed at the SARS-CoV-2 nucleocapside) and the SARS-CoV-2 IgG II Quant (Abbott, Rungis, France) for the quantitative detection of anti-S IgG (aimed at the SARS-CoV-2 spike protein). All dosages were performed on the AlinityI platform (Abbott, Rungis, France), according the manufacturer’s recommendations.

We also analyzed routine serological assessments of prioritized health workers who were vaccinated in the same hospital, as a control cohort for patients with hematologic malignancies (although they were mostly older in age) but to facilitate comparison of vaccine immunogenicity.

### Neutralization experiments

A SARS-CoV-2 surrogate neutralization assay, based on antibody-mediated blockade of ACE-2-Spike protein interaction was used, according to the manufacturer’s recommendations (ichromax Covid-19 nAB, Boditech, South Corea). Briefly, the SARS-CoV-2 neutralizing antibodies (nABs) present in sera samples were preincubated with a fluorescence-labeled SARS-CoV-2 Spike RBD antigen, in a detection buffer containing ACE-2-biotin conjugate. The mixture was then loaded in a lateral flow nitrocellulose matrix where covalent complexes RBD-ACE-2-biotin are immobilized on the streptavidine capture “Test line”. The more nAbs is present, the more it interferes with the binding of labeled RBD to ACE-2-biotin, which results in less fluorescence signal. A fluorescence inhibition above 30% is considered as positive. This semi-quantitative assay both correlates with a neutralizing SARS-CoV-2 Ab ELISA assay (Boditech Package insert) and whole-virus neutralization assay in cell culture (Marot et al.; manuscript in preparation).

### T-cell response assessment

IFN-γ production was measured on peripheral blood mononuclear cells (PBMC) obtained from excess blood available from routine laboratory tests performed in these patients as part of their standard care, before vaccination and after the second dose, using IFN-γ ELISpots kits (Diaclone, France) according to the manufacturers’ instructions. After thawing, 3 × 10^5^ PBMC were added to the wells in X-VIVO 15 medium (Lonza, Basel, Switzerland), and stimulated with peptide pools spanning the SARS-CoV-2 spike protein (pool 1 and pool 2) and the receptor binding domain (RBD), membrane protein (VME1), or CEF (CMV, EBV, Influenza virus, MHC class I epitopes) + CEFT (CMV, EBV, Influenza virus, Tetanus toxoid, MHC class II epitopes) as positive controls (PepMix from JPT peptides, final concentration 1 μg/mL). Medium containing DMSO was used as the negative control. Assays were performed in duplicate and incubated for 18–20 h at 37 °C with 5% CO_2_. ELISpot plates were counted using the C.T.L Immunospot system (Cellular Technology Limited, Cleveland, Ohio, United States of America) and assessed with Immunospot 5.0 analyser software. Values were expressed as spot forming cells/10^6^ PBMC and calculated after subtraction of values from negative-control wells. T-cell responses were considered as positive when the spot number was ≥10 and ratio 2.5-fold above background.

### Statistical analysis

The Mann–Whitney nonparametric test was used to compare anti-S IgG level or T-cell responses [receptor binding domain (RBD), entire S protein, VME1 and CEF/CEFT] according to clinical [age, body mass index (BMI), patients’ gender, diagnosis and treatment status] or biological (CD19^+^ B cells and CD3^+^ T cells) parameters. The nonparametric Wilcoxon paired test was used to compare anti-S IgG or T-cell response levels at day 0 versus day 42. Correlations between anti-S IgG and CD19^+^ B-cell and T-cell responses were computed using nonparametric Spearman correlation. Generalized Maximally Selected Statistics was used to determine the best cutoff point for day 42 anti-S IgG levels that could predict achievement of seroneutralization. Multivariate regression analyses were performed to identify clinical and/or biological parameters associated with humoral and cellular response to BNT162b2. Statistical analyses were performed with GraphPad Software (GraphPad Prism 9, San Diego, CA) and EZR version 1.27 (Saitama Medical Center, Jichi Medical University), a graphical user interface for R (The R Foundation for Statistical Computing; version 3.1.1).

## Results

### Population characteristics

**Fig. 1:**
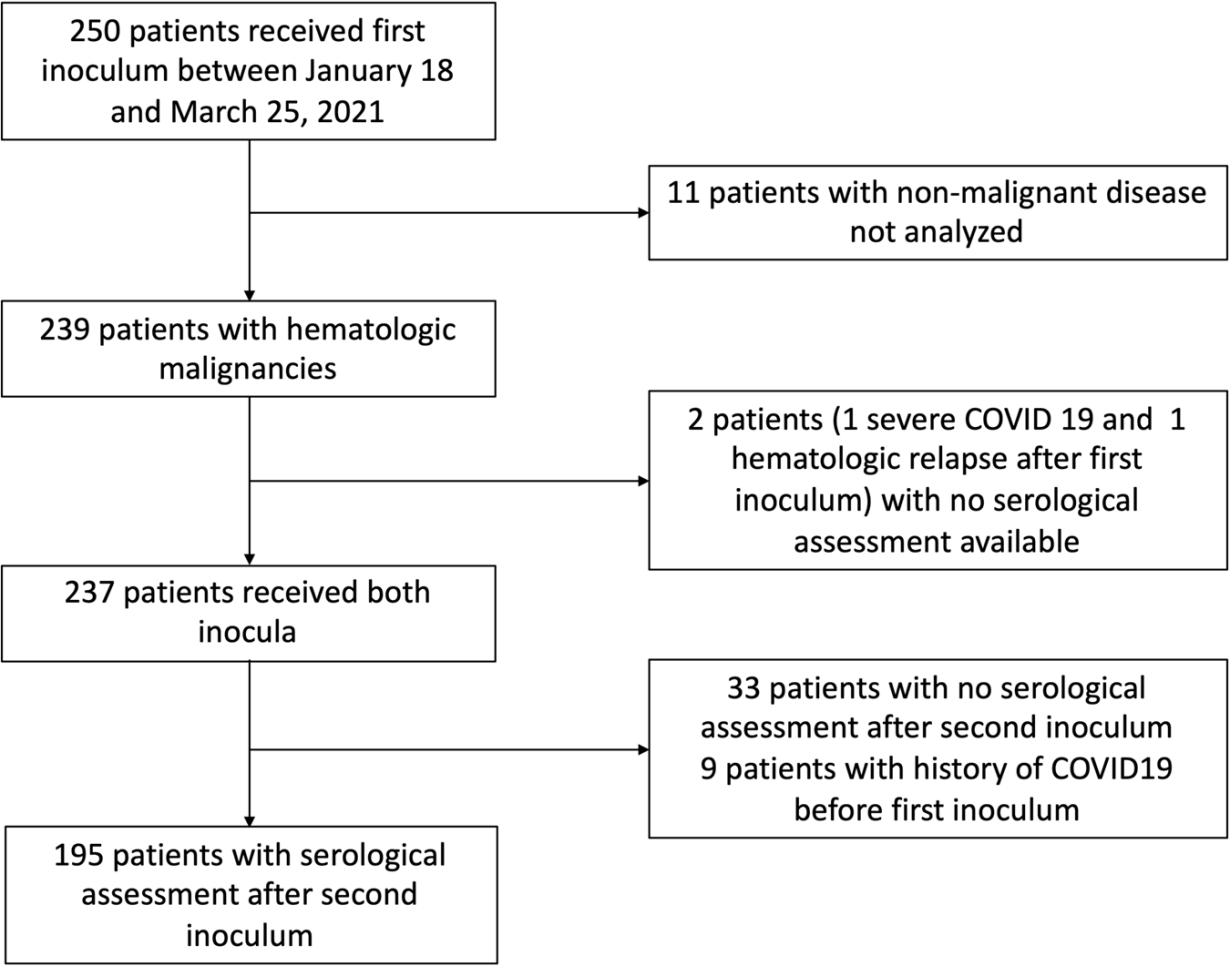
Patients’ population. Patients with hematological malignancies assessed for response after vaccination with the BNT162b2 vaccine.

In total, 239 patients with a hematologic malignancy received their first vaccination between January 18 and March 25, 2021 (Fig. 1), of which, 237 received a second dose 28 days later. One patient was not able to receive the second injection due to contracting COVID-19 in the meantime, and one patient due to disease progression and death from the underlying hematologic malignancy. Thirty-three patients had no serological assessment after the second BNT162b2 inoculum and were not analyzed. Finally, since a history of COVID-19 has been associated with an enhanced response to the COVID-19 vaccine [11], five patients with a medical history of COVID-19 before vaccination and four with no history but positive for anti-N IgG antibodies at baseline were analyzed separately. Overall, we were able to evaluate the immune-efficacy of two injections of BNT162b2 in 195 patients (Table 1). Their median age was 68.9 (range, 21.5–91.7) years. The most common diagnoses were multiple myeloma (*n* = 52) and non-Hodgkin lymphoma (*n* = 44); 136 patients had a lymphoid and 59 a myeloid malignancy. Most patients had a history of chemotherapy (*n* = 161, 82.6%) including 67 with ongoing treatment. In addition, 82 patients had a history of HCT or CAR T-cell treatment. Finally, the majority of patients (*n* = 130, 66.7%) had a history of B-cell depletion treatment, including 57 (29.2%) who received such treatment at the time of vaccination or within the past 12 months.

**Table 1:** Patients’ characteristics.

Category	Patients’ cohort *N* = 195
Median age, years (range)	68.9 (21.5–91.7)
Male	117 (60.0%)
Female	78 (40.0%)
BMI	24.8 (17.8–61.8)
Lymphoid malignancies	136 (69.7%)
Acute lymphoblastic leukemia	3 (1.5%)
Non-Hodgkin lymphoma	44 (22.6%)
Hodgkin lymphoma	5 (2.6%)
Chronic lymphocytic leukemia	26 (13.3%)
Multiple myeloma	52 (26.7%)
MGUS	6 (3.0%)
Myeloid malignancies	59 (30.3%)
Acute myeloid leukemia	31 (15.9%)
Myelodysplastic syndrome	10 (5.1%)
Myeloproliferative neoplasm	18 (9.2%)
Current status	
Untreated	34 (17.4%)
Stable disease	9 (4.6%)
Progressive disease	8 (4.1%)
Partial response	18 (9.2%)
Complete response	127 (65.1%)
History of chemotherapy	161 (82.6%)
Previous chemotherapy	94 (48.2%)
Ongoing chemotherapy	67 (34.4%)
History of HCT/cell therapy	82^a^ (42.1%)
Autologous HCT	41 (21.0%)
Allogeneic HCT	41 (21.0%)
CAR T-cells	1 (0.5%)
B-cell-targeted therapy^b^	
Yes	130 (66.7%)
Within the last 12 months	57 (29.2%)
>12 months	73 (37.4%)
Anti-CD20 monoclonal antibody	54 (27.7%)
Venetoclax	4 (2.1%)
Ibrutinib	3 (1.4%)
Cladribine	2 (2.0%)
Campath	1 (0.5%)
Anti-CD19 CAR T-cells	1 (0.5%)
Allo-HCT conditioning regimen	21 (10.8%)
IMiD/proteasome inhibitor/anti-CD38 monoclonal antibody	44 (22.6%)

*BMI* is for body mass index, *MGUS* monoclonal gammopathy of undetermined significance, *HCT* hematopoietic cell transplantation, *IMiD* immunomodulatory imide drugs.

^a^One patient received autologous and allogeneic HCT.

^b^Only the latest B-cell targeted therapy is considered.

### BNT162b2 safety and tolerability

After the first injection, 57.1% (*n* = 88) of patients developed adverse events, all of them being grade 1–2 (Supplementary Fig. 1, *n* = 154). The most common were injection site pain (42.9%), fatigue (20.1%), and myalgia (10.4%). After the second injection of BNT162b2, 34.4% of patients showed adverse events (grade 1–2, 26%; grade 3, 8.4%; grade 4, 0%), of the same most common types: injection site pain (grade 1–2, 23.4%; grade 3, 1.9%), fatigue (grade 1–2, 13%; grade 3, 5.8%), and myalgia (grade 1–2, 13%; grade 3, 3.9%).

### Serological immune response

**Fig. 2:**
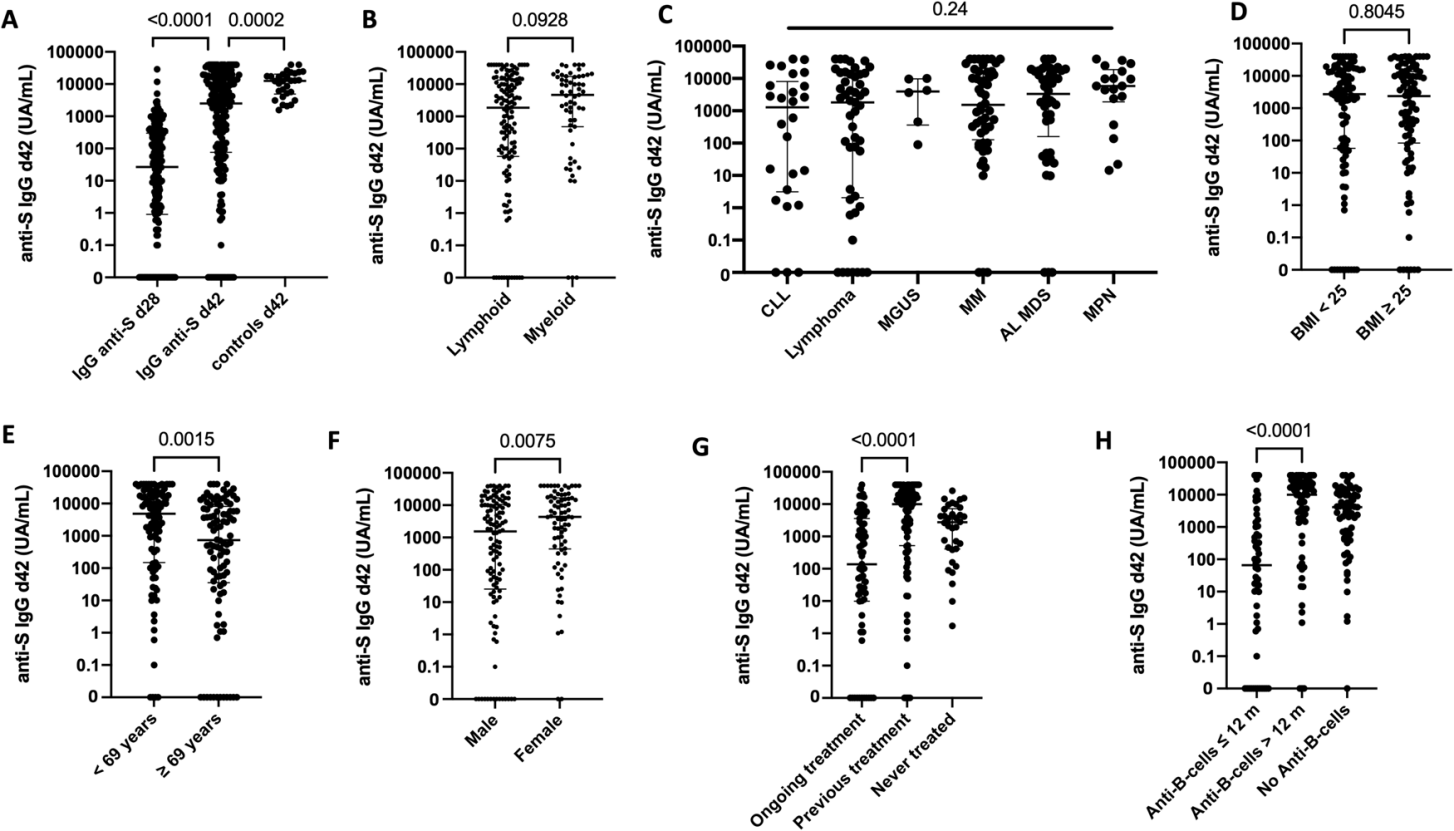
Serological response to COVID-19 BNT162b2 vaccine. Anti-S IgG antibodies were evaluated before the second BNT162b2 vaccine dose at day 28 (d28) and 2 weeks later at day 42 (d42). **A** anti-S IgG antibody at d28 and d42 in patients (*n* = 195) and at d42 in healthy controls (*n* = 30). **B–H** anti-S IgG antibody at d42 according to disease (**B–C**), body mass index (**D**), patients age (**E**), gender (**F**), treatment status (**G**), and use of anti-B-cells treatment (**H**).

In the 195 patients analyzed, we observed a significant increase of anti-S IgG antibodies between day 28 and day 42 (*p* < 0.0001, Fig. 2A). Nevertheless, compared to healthy controls (*n* = 30), anti-S IgG at day 42 were significantly lower in patients with hematologic malignancies (*p* = 0.0002, Fig. 2A). We, then, sought to assess factors associated with antibody titer. Body mass index and underlying disease had no impact on anti-S IgG at day 42 (Fig. 2B-D). In contrast, male and/or older patients, those with ongoing chemotherapy and those receiving anti-B-cell treatment within the previous 12 months had significantly lower anti-S IgG at day 42 (Fig. 2E-H).

### Factors predicting serological immune response and neutralization

**Fig. 3:**
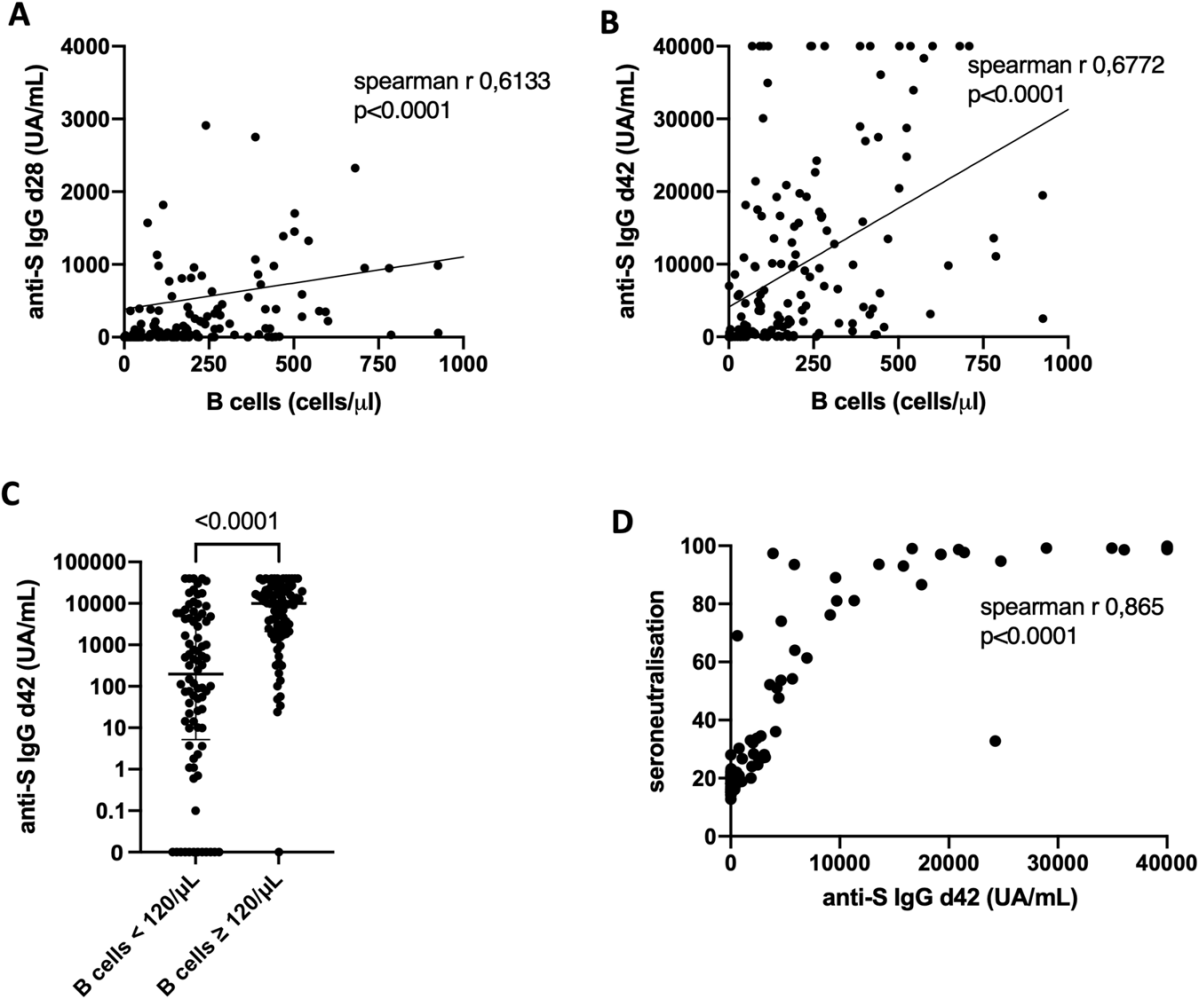
Correlation between humoral response and B cells and seroneutralization. **A, B** Correlation between anti-S IgG at d28 (**A**) and d42 (**B**) and CD19^+^ B cells (*n* = 174), patients with circulating pathologic B cells (chronic lymphocytic leukemia not in complete remission and some non-Hodgkin lymphoma patients) were excluded from this analysis. **C** anti-S IgG antibody at d42 according CD19^+^ B-cells level. **D** Correlation between serum neutralization and anti-S IgG antibodies at d42 (*n* = 97).

Since B cells are the drivers of serological response, we evaluated the relationship between CD19^+^ B cells and anti-S IgG at day 28 and day 42. We found strong correlations between the two parameters both on day 28 (Spearman r = 0.613, *p* < 0.0001; Fig. 3A) and day 42 (Spearman r = 0.677, *p* < 0.0001; Fig. 3B), indicating that B cells are likely the main driver of immune response after BNT162b2 inoculation. Finally, patients with B cells <120 /μL had a significantly lower anti-S IgG level at day 42 (*p* < 0.0001; Fig. 3C). Importantly, there was no correlation between anti-S IgG levels at day 42 and total gamma globulin levels, total lymphocyte, CD3^+^ T cell, CD4^+^ T cell, or CD8^+^ T cell counts (data not shown).

The threshold for anti-S IgG positivity in being protective against COVID19 has not yet been defined. However, recent evidences have linked the clinical efficacy of immune response to the neutralizing antibody levels in serum of infected or vaccinated patients [12]. Therefore, we evaluated neutralizing antibodies (NAb) in 97 patients using excess serum available from other routine laboratory tests performed in these patients as part of their standard care. We found that NAb correlated with anti-S IgG day-42 titers (Spearman r = 865, *p* < 0.0001; Fig. 3D). Furthermore, we found that an anti-S IgG day-42 level ≥3100 UA/mL was predictive of a NAb ≥ 30%, the positivity cutoff for NAb (*p* < 0.0001, Supplementary table 1). Therefore, we defined an anti-S IgG day-42 level ≥3100 UA/mL as the threshold for a neutralizing and protective response. In our cohort, only 3/195 patients (1.5%) achieved this level at day 42 after the first BNT162b2 inoculum and 91/196 (46.7%) after second inoculum, compared to 26/30 (87%) of healthy controls.

We then performed a logistic regression to identify in a multivariate analysis, parameters associated with achievement of protective anti-S IgG day-42 level (≥3100 UA/mL). We found that patients’ age (≥71 years versus <71 years), BMI (>25 versus ≤25 kg/m^2^), and CD19^+^ B-cell level (<120/μL versus ≥120/μL) had no impact on achievement of a protective anti-S IgG level after two BNT162b2 inoculum. In contrast, male gender and ongoing chemotherapy were associated with a significantly decreased probability of achieving the defined protective anti-S IgG level after two BNT162b2 inoculum [odds ratio (OR) 0.126, 95% confidence interval (95CI) 0.022–0.709, *p* = 0.02; OR 0.146, 95% CI 0.025–0.866, *p* = 0.03 respectively].

### T-cell response

**Fig. 4:**
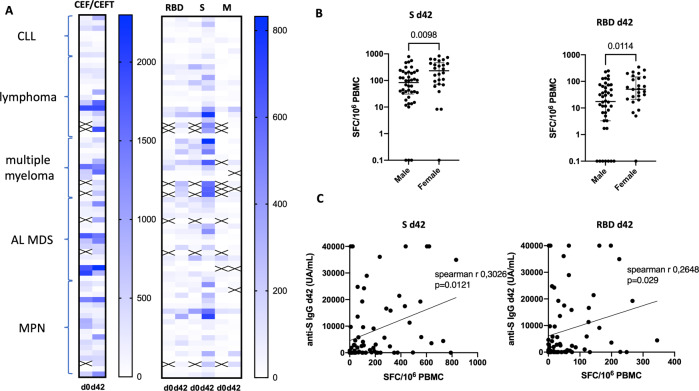
T-cell response to COVID-19 BNT162b2 vaccine. **A** T-cell response was assessed by IFN-γ ELISPOT at baseline (before first BNT162b2 vaccination, d0) and 2 weeks after the second BNT162b2 vaccination (d42), *n* = 68. T-cell response against CMV, EBV, Influenza virus, and Tetanus toxoid (CEF/CEFT, left panel). T-cell response against SARS-CoV-2 spike protein (S), or its receptor binding domain (RBD), or against membrane protein (M) (right panel). Cross indicates missing data. CLL chronic lymphocytic leukemia, AL MDS acute leukemia and myelodysplastic syndrome, MPN myeloproliferative neoplasm. **B** T-cell response against S and RBD at d42 according to patients’ gender. **C** Correlation between T-cell responses against S and RBD and anti-S IgG at d42.

Finally, we assessed T-cell response in 68 patients with available PBMC at baseline (before first vaccine) and/or after second vaccine dose (day 42), using the ELISPOT assay. We found a significant increase in T-cell response directed against the S protein (either the RBD or the entire S protein) targeted by the vaccine, while there was no increase against the membrane protein (VME1) or a pool of viral peptides (CMV, EBV, and Flu) used as controls (CEF/CEFT) (Fig. 4A and Supplementary Fig. 2). Therefore, 53% of patients (*n* = 36) had a cellular response against the S protein after the second BNT162b2 inoculum.

Next, we sought to assess factors associated with T-cell response. Underlying disease, patient’s age, BMI, treatment status, use of anti-B-cell treatment within the previous 12 months and CD3^+^ T cells had no impact on T-cell response (data not shown), while male gender was associated with a decreased T-cell response (*p* = 0.098 for the entire S protein and *p* = 0.011 for the RBD, Fig. 4B). Furthermore, there was no correlation between T-cell response against the S protein and the number of the CD3^+^, CD4^+^, or CD8^+^ T cells (data not shown). However, we observed a correlation with the anti-S IgG day-42 level (Spearman r = 0.3026, *p* = 0.012 for the entire S protein and spearman r = 0.2648, *p* = 0.029 for the RBD; Fig. 4C). Interestingly, among the 36 patients with a T-cell response directed against the S protein, 17 did not achieve a protective anti-S IgG level (≥ 3100 UA/mL) after the second BNT162b2 inoculum, including 15 with impaired B-cell status (B-cell lymphopenia, *n* = 12 or active CLL *n* = 3), suggesting that immunity to vaccine was not completely abrogated in these patients. Overall, in patients with evaluable T-cell data, 66% achieved humoral and/or cellular response after 2 BNT162b2 inocula.

Finally, a logistic regression was performed to identify parameters associated with achievement of a T-cell response against the S protein in a multivariate analysis. We found that treatment status was the only feature associated with the absence of T-cell response (current treatment versus no current treatment, OR 0.175, 95% CI 0.046–0.674, *p* = 0.011; Table 2).

**Table 2:** Multivariate analysis for humoral response and T-cell response.

Multivariate analysis for humoral response		
	Odds ratio (95% CI)	*P* value
Age (≥69 years versus <69 years)	0.529 (0.244–1.15)	0.11
Patient gender (male versus female)	0.343 (0.153–0.767)	0.009
BMI (>25 versus ≤25 kg/m^2^)	1.730 (0.803–3.710)	0.16
Diagnosis (lymphoid versus myeloid malignancy)	0.765 (0.329–1.780)	0.53
Current treatment (yes versus no)	0.646 (0.238–1.750)	0.39
Anti-B-cell treatment in the previous 12 months (yes versus no)	0.231 (0.081–0.658)	0.006
CD19^+^ B cells (≥120/μL versus <120/μL)	0.260 (0.111–0.609)	0.002

Multivariate analysis for T-cell response		
	Odds ratio (95% CI)	*P* value
Age (≥69 years versus <69 years)	0.965 (0.313–2.980)	0.95
Patient gender (male versus female)	0.476 (0.150–1.510)	0.21
BMI (>25 versus ≤25 kg/m^2^)	1.580 (0.542–4.640)	0.40
Diagnosis (lymphoid versus myeloid malignancy)	1.240 (0.339–4.510)	0.75
Current treatment (yes versus no)	0.175 (0.046–0.674)	0.011
Anti-B-cells treatment in the previous 12 months (yes versus no)	3.110 (0.713–13.500)	0.13
CD3^+^ T cells (≥850/μL versus <850/μL)	2.880 (0.889–9.320)	0.078

### Response in patients with a history of COVID-19

The humoral response was also evaluated among the nine patients with a history of COVID-19 who received two BNT162b2 inocula. Six (67%) achieved a seroprotective level of anti-S IgG at day 42 (≥3100 UA/mL). Among the nonresponders, one patient received anti-B-cell treatment (rituximab) within the previous 12 months, while the other two patients had not been treated during the same period (one untreated monoclonal gammopathy of undetermined significance and one follicular lymphoma in complete response).

## Discussion

This retrospective study evaluated safety/tolerability and immune response after 2 BNT162b2 inoculations in patients with hematologic malignancies.

The BNT162b2 vaccine was well tolerated. After the first inoculum, no patients developed grade III–IV side effects and injection site pain was the predominant complaint. Interestingly, after the second dose, while the overall incidence of side effects was lower, those of grade III were reported in 8.4% of the patients, mainly related to a flu-like syndrome (fatigue, myalgia), suggesting that enhanced immune response after the second inoculum directly contributed to the observed side effects. The overall incidence of adverse events in our cohort is comparable to that previously reported in healthy individuals in phase 1 and 2/3 clinical trials (27–50%) [3, 10] or in real life data (79%) [13]. However, while very few serious side events were reported in those studies (0–1.1%) [3, 10, 13], the incidence (8.4%) of grade 3 adverse events in our series may appear high. Nevertheless, in our patients, none of the grade 3 adverse events required hospitalization, and all recovered with self-administration of acetaminophen. Therefore, our study confirms that the BNT162b2 vaccine is safe in highly immunosuppressed patients with hematologic malignancies, and suggests that preemptive administration of acetaminophen may be recommended after the second inoculum to prevent a severe flu-like syndrome in those patients.

Regarding efficacy, previous studies reported a low seroconversion ranging from 18 to 25% after the first BNT162b2 inoculum in patients with hematologic malignancies [8, 9]. However, raw serological data are difficult to analyze since the precise threshold of anti-Spike Abs levels conferring a clinical protection and/or an effective neutralizing activity of the sera are still unknown. These correlations likely rely on the population tested as well as the serological assay used [14]. For this reason, we evaluated in our cohort the functional activity of the anti-spike antibodies quantified in routine practice by a surrogate neutralization assay based on blockade of ACE-2 Spike protein interaction. Interestingly, the best anti-spike IgG Abs cutoff level predicting an effective seroneutralization activity was quite high (3100 UA/ml), This level, which is higher than the 50 UA/ml positive cutoff given by the fabricant (Abbott) to define a positive test, was therefore used in our cohort to define serologic immunity against COVID-19 in our study. Based on this cutoff, the number of patients with seroconversion after the first BNT162b2 inoculum was only 1.5%, which is lower compared to previous reports. Nevertheless, we showed that the anti-S IgG level significantly increased after the second BNT162b2 inoculum, highlighting that a vaccine booster is effective in improving seroconversion in immunosuppressed patients. In fact, this translated into an increased number of patients with seroconversion after a second BNT162b2 inoculum, reaching 46.7% of patients. This is in accordance with preliminary data from others reporting that three out of five patients (60%) who had received two BNT162b2 inocula were seropositive [8]. We then evaluated in multivariate analysis the factors predicting seropositivity in patients with hematologic malignancies, and found that, while underlying diagnosis and patients’ age had no impact, male gender, use of anti-B-cell targeting treatment within the previous 12 months, and low CD19^+^ B-cell numbers (below the normal threshold) were associated with a failure to achieve seropositivity. Likewise, Herishanu et al. concluded that all CLL patients treated with an anti-CD20 monoclonal antibody in the previous 12 months failed to achieve seroconversion [15]. Similarly, in patients with chronic inflammatory disease, B-cell depleting agents were associated with a significantly reduced humoral response after two BNT162b2 inocula [7]. As to the reduced efficacy of BNT162b2 vaccine on males, no data are available in the setting of COVID-19. Nevertheless, it has been reported that adult females develop a much better immune response, with respect to antibody levels, and experienced more severe adverse events following immunization, due to enhanced immune activation, compared to their male counterparts [16].

Humoral immunity is clearly critical for protection against SARS-CoV-2, as evidenced by the correlations on dynamics of neutralizing antibody responses and clinical outcome [17, 18], clinical efficacy of neutralizing Abs levels [12], effectiveness of therapeutic plasma in B-cell depleted patients [19], and of monoclonal antibodies [20]. Nevertheless, cellular immunity also contributes to protection against SARS-CoV-2, and circulating SARS-CoV-2-specific CD8^+^ and CD4^+^ T cells were identified in ~70% and 100% of COVID-19 convalescent patients, respectively [21]. Therefore, in addition to humoral response, we also evaluated T-cell response to BNT162b2 using the ELISPOT assay. We found a significant increase in T-cell response to the S protein after two doses of the BNT162b2 vaccine, but not against the M protein. While the T-cell response correlates with the anti-S IgG level at day 42, some patients with B-cell lymphopenia and no humoral response achieved a T-cell response, indicating that immunity to vaccine was not completely abrogated in these patients and that T-cell response could be protective when humoral immunity is deficient. In fact, a recent study reported the crucial role of CD8^+^ T cells in contributing to the survival of patients with COVID-19 and hematologic cancer, suggesting that CD8 T cells play a key role in limiting SARS-CoV-2, even in the absence of humoral immunity [22]. These data highlight the role of T-cell responses to vaccination by providing protection in patients with hematologic cancer even in the setting of limited humoral responses. Finally, ongoing treatment was the only parameter associated with impaired T-cell response. This suggest that, while it is still mandatory to vaccinate patients under chemotherapy, vaccine response should be assessed at the end of the treatment, and additional BNT162b2 vaccination should be discussed based on the response.

The strength of this study is the comprehensive evaluation of immune response after 2 BNT162b2 inocula including functional seroneutralization assay and assessment of T-cell response. We were therefore able to accurately evaluate the patients’ protective seroconversion against COVID-19 and identify factors predictive of failure. One could suggest that our study lacks an age-matched control group. nevertheless, the aim was to evaluate BNT162b2 vaccine outcomes among patients with hematologic malignancies and to identify factors for poor immune response in this specific population.

Overall, our findings suggest that a second BNT162b2 inoculum translates into a significant increase in humoral response, allowing almost half of the patients to achieve immune protection against COVID-19. Nevertheless, the use of B-cell targeting treatment within the previous 12 months before vaccination, and a low CD19^+^ B-cell level predict failure to achieve immune protection. While some data may suggest delaying COVID-19 vaccination beyond 12 months after use of a B-cell depleting agent and B-cell recovery, health authorities now recommend the administration of a third COVID-19 inoculum in immunosuppressed patients. Given the increase in seroconversion rate between the first and second vaccine injections, evaluation of the effectiveness of such a third inoculum will be imperative.

## Supplementary information

Supplementary file

Checklist
