# Supplementary material for: Weak immunogenicity of SARS-CoV-2 vaccine in patients with hematologic malignancies
Source: Blood Cancer J. 2021 Aug 10;11(8):142. doi: 10.1038/s41408-021-00534-z (PMC8353615; doi:10.1038/s41408-021-00534-z)

Weak immunogenicity of SARS-CoV-2 vaccine in patients with hematologic malignancies.

Florent Malard[1,2*], Béatrice Gaugler[1,2*], Joel Gozlan[2,3*], Lucie Bouquet[4], Djeneba Fofana[3,5], Lama Siblany[1,2], Deborah Eshagh[2], Olivier Adotevi[4], Caroline Laheurte[4], Laure Ricard[1,2], Rémy Dulery[1,2], Nicolas Stocker[1,2], Zoe van de Wyngaert[1,2], Alexis Genthon[1,2], Anne Bannet[1,2], Mara Memoli[1], Souhila Ikhlef[1], Simona Sestilli[1,2], Anne Vekhof[1], Eolia Brissot[1,2], Zora Marjanovitch[1], Yannick Chantran[6], Nancy Cuervo[3], Eric Ballot[6], Laurence Morand-Joubert[3,5], Mohamad Mohty[1,2#]

[1] APHP, Hôpital Saint Antoine, Service d'Hématologie Clinique et de Thérapie cellulaire, Paris, France

[2] Sorbonne Université, INSERM UMR938, Centre de Recherche Saint-Antoine (CRSA), F-75012 Paris, France

[3] APHP, Hôpital Saint Antoine, Department of Virology, Paris, France

[4] Department of Medical Oncology, University Hospital of Besançon, INSERM, EFS BFC, UMR1098 RIGHT, Université de Bourgogne Franche-Comté, France

[5] Sorbonne Université Institut Pierre Louis d'Epidémiologie et de Santé Publique, INSERM UMR S1136

[6] APHP, Hôpital Saint Antoine, Service d'Immunologie, Paris, France

* These authors contributed equally to this work and should be considered as first authors.

Supplementary file

Supplementary Table 1.

		inhibition		Total
		<30%	>=30%	
**Anti-S IgG**	<3100	56 (98.2%)	6 (15.0%)	62
	>=3100	1 (1.8%)	34 (85%)	35
	Total	57	40	97

Supplementary Figure 1. Adverse events.
Adverse events after first (A) and second (B) inoculum of BNT162b2 vaccine.

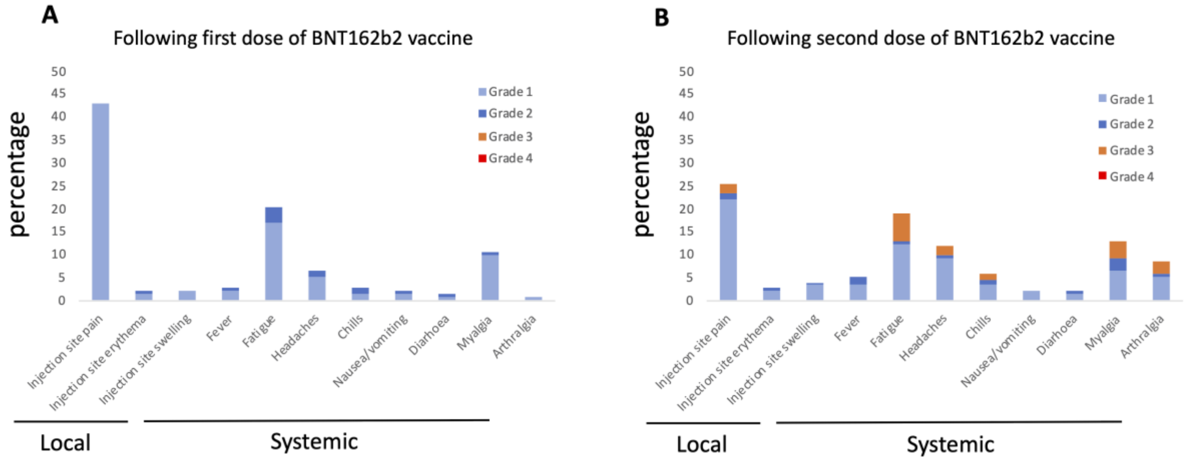

Supplementary Figure 2. T cells response to COVID-19 BNT162b2 vaccine.
T cell response was assessed by IFN-γ ELISPOT at baseline (before first BNT162b2 vaccination, d0) and 2 weeks after the second BNT162b2 vaccination (d42), n=68. T cell response against CMV, EBV, Influenza virus and Tetanus toxoid (CEF/CEFT). T cell response against SARS-CoV-2 spike protein (S), or its receptor binding domain (RBD), or against membrane protein (M). CLL is for chronic lymphocytic leukemia; AL MDS, acute leukemia and myelodysplastic syndrome; MPN, myeloproliferative neoplasm.

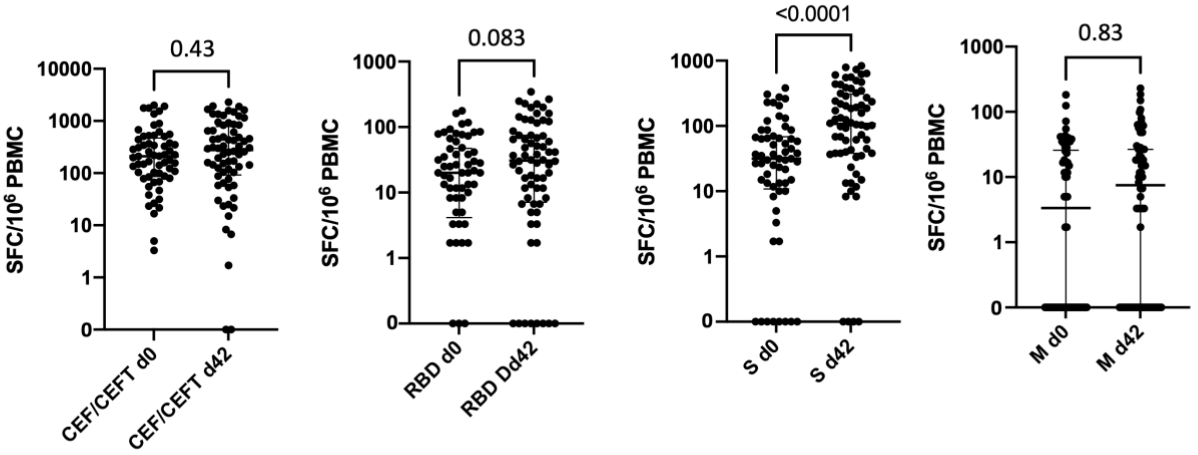

Supplement: Supplementary file 1 — Supplementary file [file 41408_2021_534_MOESM1_ESM.pdf]
